# A retrospective population based cohort study of access to specialist palliative care in the last year of life: who is still missing out a decade on?

**DOI:** 10.1186/s12904-016-0119-2

**Published:** 2016-05-10

**Authors:** Lorna Rosenwax, Katrina Spilsbury, Beverley A. McNamara, James B. Semmens

**Affiliations:** School of Occupational Therapy and Social Work, Faculty of Health Sciences, Curtin University, GPO Box U1987, Perth, 6845 Australia; Centre for Population Health Research, Faculty of Health Sciences, Curtin University, Perth, Australia

**Keywords:** Palliative care, Population-based study, Community based palliative care, Hospital-based palliative care, Cancer, Non-cancer conditions, Life limiting illness

## Abstract

**Background:**

Historically, specialist palliative care has been accessed by a greater proportion of people dying with cancer compared to people with other life-limiting conditions. More recently, a variety of measures to improve access to palliative care for people dying from non-cancer conditions have been implemented. There are few rigorous population-based studies that document changes in palliative care service delivery relative to the number of patients who could benefit from such services.

**Method:**

A retrospective cohort study of the last year of life of persons with an underlying cause of death in 2009–10 from cancer, heart failure, renal failure, liver failure, chronic obstructive pulmonary disease, Alzheimer’s disease, motor neurone disease, Parkinson’s disease, Huntington’s disease and/or HIV/AIDS. The proportion of decedents receiving specialist palliative care was compared to a 2000–02 cohort. Logistic regression models were used identify social and demographic factors associated with accessing specialist palliative care.

**Results:**

There were 12,817 deaths included into the cohort; 7166 (56 %) from cancer, 527 (4 %) from both cancer and non-cancer conditions and 5124 (40 %) from non-cancer conditions. Overall, 46.3 % of decedents received community and/or hospital based specialist palliative care; a 3.5 % (95 % CI 2.3–4.7) increase on specialist palliative care access reported ten years earlier. The majority (69 %; *n* = 4928) of decedents with cancer accessed palliative care during the last year of life. Only 14 % (*n* = 729) of decedents with non-cancer conditions accessed specialist palliative care, however, this represented a 6.1 % (95 % CI 4.9–7.3) increase on the specialist palliative care access reported for the same decedent group ten years earlier. Compared to decedents with heart failure, increased odds of palliative care access was observed for decedents with cancer (OR 10.5; 95 % CI 9.1–12.2), renal failure (OR 1.5; 95 % CI 1.3–1.9), liver failure (OR 2.3; 95 % CI 1.7–3.3) or motor neurone disease (OR 4.5; 95 % CI 3.1–6.6). Living in major cities, being female, having a partner and living in a private residence was associated with increased odds of access to specialist palliative care.

**Conclusion:**

There is small but significant increase in access to specialist palliative care services in Western Australia, specifically in patients dying with non-cancer conditions.

**Electronic supplementary material:**

The online version of this article (doi:10.1186/s12904-016-0119-2) contains supplementary material, which is available to authorized users.

## Background

Specialist palliative care has been shown to improve symptom management, reduce hospitalizations, allow patients to be safely cared for at home and result in improved patient and family satisfaction for both cancer and non-cancer patients. Palliative care also lowers costs by reducing acute care health service use [1]. One area that remains a concern is the number of people with life limiting non-cancer conditions who do not access specialist palliative care. Despite early calls to develop, fund and evaluate appropriate cost effective services to meet the needs of patients with advanced non-cancer diseases [2, 3], there is little evidence to demonstrate significant progress. A 2014 Spanish study that estimated the prevalence of patients in need of palliative care reported a ratio of cancer to non-cancer patients as 1:7, although palliative care service delivery was not measured [4]. A German study from around the same time reported that inpatient palliative care services were being delivered to an increasing proportion of patients for non-cancer patients; an increase from 3.5 to 8.1 % over a four year period [5]. Unfortunately there are few rigorous population-based studies that documented changes in both community based and hospital based palliative care service delivery relative to the number of patients who could benefit from such services.

It is well established that people with non-cancer conditions do not access specialist palliative care as readily. Definitions of palliative care rarely refer to specific diseases. The World Health Organisation uses the terminology “life threatening illness” in defining who should receive palliative care [6]. Models of palliative care and gold standard frameworks invariably propose the provision of services to people with life limiting illness, regardless of diagnosis [7–9]. A study using population-based estimates refined by expert panel review argued that palliative care must be extended to non-cancer conditions and that between 69 and 82 % of people who die need palliative care [10]. Clinical evidence demonstrates that there are commonalities in the prevalence of problems across cancer and non-cancer conditions [11, 12]. However, the illness trajectories of non-cancer diseases may be longer and have unpredictable fluctuations [13] both of which complicate the provision of cost-effective specialist palliative care service. Other complex factors such as patient and family awareness and preparedness also impact the provision of palliative care to people with non-cancer conditions [14].

Without significant progress towards the provision of palliative care to people dying with non-cancer conditions, we are not meeting the needs of those who could benefit from palliative care under contemporary definitions. Data provided at a population level greatly assists the delivery of health services and can be used to promote greater access to palliative care for people dying of non-cancer conditions. This paper uses population-based Australian data to examine trends in palliative care provision across a ten-year period. The study investigates whether people dying of non-cancer conditions are getting greater access to specialist palliative care, given current definitions and clinical evidence to support the need for such care. It also explores other socio-demographic inequities in order to highlight areas of under-developed specialist palliative care service provision. There is ongoing concern that older people [15] and people living in rural areas [16, 17] may be missing out on specialist palliative care. Drawing on an earlier Australian study which defined conditions considered potentially amenable to palliative care [18], this study traces specialist palliative care service use in the last year of life by accessing linked mortality, hospital morbidity and community nursing and medical services records.

## Methods

### Study design

This was a retrospective cohort study of the last year of life of persons who had an underlying cause of death potentially amenable to receiving palliative care. A de-identified and linked extraction of death records, hospital morbidity records and community-based care records of persons who died in Western Australia (WA) from 1st January 2009 to 31st December 2010 was obtained from the Data Linkage Branch at the WA Department of Health. The data extraction also included geo-coded and type of residence information based on decedent’s residential address at time of death and at each hospital admission. Ethical approval to conduct this study was provided by Human Research Ethics Committees at the WA Department of Health and Curtin University.

### Cohort selection

The cohort was identified by any mention of one or more of the 10 disease conditions considered amenable for palliative care as defined by Rosenwax et al. [18] coded anywhere in Part I (underlying cause of death) of the death certificate. The 10 disease conditions were cancer, heart failure, renal failure, liver failure, chronic obstructive pulmonary disease, Alzheimer’s disease, motor neurone disease, Parkinson’s disease, Huntington’s disease and HIV/AIDS. The International Classification of Disease (ICD)-10-Australian Modification codes used to identify the disease conditions are supplied as Additional file 1: Table S1.

For decedents with more than one of the disease conditions mentioned on Part 1 of the death certificate, a hierarchical approach based on the death certification concept of underlying cause was used to classify the principal disease condition [19]. For example, a decedent with liver failure listed as the direct cause of death but who also had carcinoma of pancreas recorded as the underlying antecedent cause was assigned as having cancer as the principal disease condition amenable to palliative care. We note that using these classification rules meant that the principal disease condition did not always match the final single underlying cause of death. For example, a decedent with heart failure listed as a direct cause of death may have had atrial fibrillation listed as the final underlying cause of death but was still classified as heart failure being the principal disease condition amenable to palliative care for the purposes of this study. Information from Part II of the death certificate (other significant conditions contributing to death but not related to the condition causing it) was not used to classify disease conditions amenable to palliative care here.

To directly compare findings here with those of a study performed ten years earlier [3], the disease conditions considered amenable to palliative care in each decedent were further grouped into being cancer conditions only, cancer and non-cancer conditions or non-cancer conditions.

### Defining specialist palliative care

Specialist palliative care was defined as receiving specific community-based or hospital-based palliative care. Community-based care data was provided by Silver Chain WA, a not-for-profit organisation that provides over 90 % of all in-home health and care and 100 % of in-home palliative care in WA [20]. Silver Chain community-based palliative care is only provided with a referral from a medical practitioner and includes at-home physical care and practical support, symptom management (for example pain, nausea), counselling and respite care. This community-based palliative care may occur in private residences or in non-hospital care facilities and was mostly restricted to major urban areas and some country areas.

Hospital-based palliative care in WA is classified as care in a hospital palliative care unit, in a designated palliative care program or under the principal clinical management of a palliative care physician or when the clinical intent of care is palliation. Hospital-based palliative care was identified from hospital morbidity records where the episode of care was coded as palliative and can be provided by most major metropolitan, country hospitals and both private and public hospices [21].

### Social and demographic variables

Marital status at time of death was classified as partnered (married or de-facto) or not/unknown. Geocoding of decedents address at time of death was used to assign accessibility to services categories that were based on the ARIA+ index that takes into account road distance measurements to the nearest Service Centres and population size [22]. Similarly, geocoding was used to assign quintiles of the Index of Relative Social Disadvantage [23]. Residences at time of death were classified as private residence, residential aged care facility, non-aged care facilities, or other/unknown.

### Statistical analysis

Data were structured so that every day of the last year of life for each decedent was assigned to a care state. Care states were classified as usual care, community-based non-palliative care, community-based palliative care, hospital non-palliative care and hospital palliative care. Decedents who were residing in hospital were classified as receiving hospital non-palliative care. Fishers’ exact tests were used to assess equality of proportions including comparing proportions of decedents receiving specialist palliative care with those reported ten years earlier [3]. Logistic regression models were used to assess the strength of association between accessing specialist palliative care and various social, demographic and cause of death disease conditions. Hosmer-Lemeshow goodness of fit tests were used to evaluate model specification and plausible interaction terms tested. Data manipulation and analysis were performed using Stata v13 (College Station, TX).

## Results

There were 23,852 non-trauma and non-obstetric related deaths in Western Australia in 2009–2010 and of these, 12,817 (53.7 %) deaths met the criteria for inclusion into the cohort of the 10 disease conditions considered as being amenable to receiving palliative care. There were 1,052 (8.2 %) decedents with more than one disease conditions listed on Part 1 of the death certificate. Median age at death was 78 years (Interquartile range 67–86 years) and ranged from 20 years to 107 years of age. The mean age at death was slightly younger at 75.7 years (Standard Deviation 13.5 years) indicating age was negatively skewed. Socio-demographic characteristics of the cohort are summarised in Table 1 by whether the principal cause of death conditions included cancers or not. Sixty percent of the cohort had cancer mentioned on Part 1 of the death certificate with a small proportion (6.9 %) of these decedents also having other disease conditions considered amenable to palliative care listed. In general, decedents with cancer were younger, more likely to have a partner and more likely to be living in a private residence at time of death.

**Table 1:** Summary characteristics of the cohort at time of death by cause of death category (*n* = 12817)

Socio-demographic variables	Cancer only		Cancer + non cancer		Non-cancer only		All	
	*n* = 7166		*n* = 527		*n* = 5124		*n* = 12817	
	No.	%	No.	%	No.	%	No.	%
Age at death (years)								
< 60	1,262	17.6	72	13.7	322	6.3	1,656	12.9
60–69	1,527	21.3	83	15.7	402	7.8	2,012	15.7
70–79	2,007	28.0	167	31.7	1,035	20.2	3,209	25.0
80–89	1,904	26.6	175	33.2	2,138	41.7	4,217	32.9
90+	466	6.5	30	5.7	1,227	23.9	1,723	13.4
Sex								
Male	4,090	57.1	333	63.2	2,521	49.2	6,944	54.2
Female	3,076	42.9	194	36.8	2,603	50.8	5,873	45.8
Partnered at death								
No	3,279	45.8	260	49.3	3,322	64.8	6,861	53.5
Yes	3,887	54.2	267	50.7	1,802	35.2	5,956	46.5
Accessibility of residence								
Major cities	4,958	69.4	355	67.7	3,471	68.0	8,784	68.7
Inner regional	1,184	16.6	88	16.8	848	16.6	2,120	16.6
Outer regional	663	9.3	51	9.7	502	9.8	1,216	9.5
Remote	238	3.3	20	3.8	170	3.3	428	3.3
Very remote	106	1.5	10	1.9	117	2.3	233	1.8
IRSD of area of residence								
Most disadvantage	1,464	20.5	132	25.2	1,192	23.3	2,788	21.8
More disadvantage	1,571	22.0	99	18.9	1,110	21.7	2,780	21.8
Average disadvantage	1,434	20.1	115	21.9	1,056	20.7	2,605	20.4
Less disadvantage	1,265	17.7	89	17.0	822	16.1	2,176	17.0
Least disadvantage	1,415	19.8	89	17.0	928	18.2	2,432	19.0
Residence type at death								
Private	6,393	89.2	438	83.1	2,963	57.8	9,794	76.4
RACF	684	9.5	77	14.6	1,962	38.3	2,723	21.2
Other care facility	43	0.6	5	0.9	109	2.1	157	1.2
Unknown/NFA/other	46	0.6	7	1.3	90	1.8	143	1.1

*RACF* residential aged care facility, *NFA* no fixed address, *IRSD* Index of relative social disadvantage

### Use of hospital and community based palliative care in last year of life

Overall, 46.3 % of decedents received at least one day in the last year of life under community and/or hospital based specialist palliative care (Table 2). This represents a 3.5 % (95 % CI 2.3–4.7; *p*-value <0.0001)) increase on specialist palliative care access reported ten years earlier [3]. Specialist palliative care in the last year of life was accessed by 69 % of decedents with cancer only, with almost half (48 %) of cancer decedents accessing palliative care via the community. There was no significant change in the proportion of decedents with cancer who accessed specialist palliative care over the last ten years (*p*-value 0.182). Around 20 % of decedents with cancer only accessed palliative care through a hospital only. For cancer decedents who also had other cause of death conditions recorded on the death certificate, access to specialist palliative care was reduced compared to those with cancer only.

**Table 2:** The number and proportion of cohort who accessed specialist palliative care in the last year of life (*n* = 12817)

Type of specialist palliative care received										
		Community only		Hospital only		Community and hospital				
	All No.	*n* = 2270		*n* = 1986		*n* = 1676		Total specialist palliative care		
Cause of death		No.	%	No.	%	No.	%	No.	%	%Δ^a^
Grouped										
Cancer only	7166	1,907	26.6	1,484	20.7	1,537	21.4	4,928	68.8	+1.0
Cancer + non cancer	527	120	22.8	90	17.1	65	12.3	275	52.2	+4.0
Non cancer only	5124	243	4.7	412	8.0	74	1.4	729	14.2	+6.1*
Total	12817	2270	17.7	1986	15.5	1676	13.1	5,932	46.3	+3.5*
Principal										
Cancer	7411	1,967	26.5	1,522	20.5	1,5651	21.1	5,054	68.2	+1.0
Heart failure	2019	94	4.7	148	7.3	23	1.1	265	13.1	+3.8*
Renal failure	1145	63	5.5	135	11.8	27	2.4	225	19.7	+8.1*
COPD	1094	86	7.9	84	7.7	26	2.4	196	17.9	+10.7*
Alzheimer’s	608	15	2.5	22	3.6	0	0.0	37	6.1	+2.7
Liver failure	206	15	7.3	41	19.9	12	5.8	68	33.0	+16.2*
Motor neurone	136	23	16.9	17	12.5	22	16.2	62	45.6	+10.3
Parkinson’s	181	<5		15	8.3	<5		21	11.6	+7.5

*COPD* chronic obstructive pulmonary disease. Cells with less than five decedents were listed as <5

^*^Statistically significant increases with *p*-value < 0.001

^a^Percentage change in the proportion of decedents in 2009/2010 who received specialist palliative care in the last year of life compared to that reported for 2000–2002 [3, 33]

% = row percentages. Due to low numbers, subgroup analyses are not shown for decedents with HIV/AIDS (*n*<5) or Huntington's disease (*n*=13)

Specialist palliative care was accessed by 14 % of decedents with non-cancer conditions. While this is much lower than that accessed by decedents with cancer, this represents a 6.1 % (95 % CI 4.9 – 7.3; *p*-value <0.001) increase on the specialist palliative care access reported for the same decedent group ten years earlier [3]. The increase in specialist palliative care observed for non-cancer conditions occurred across both community based palliative care and hospital based palliative care. The proportion of decedents with non-cancer conditions who accessed community palliative care only increased from 3.1 to 4.7 %, and for hospital based palliative care only increased from 3.9 to 8.0 % over ten years. Decedents with motor neurone diseases and liver failure accessed specialist palliative care most frequently out of the non-cancer conditions, followed by renal failure. Those with liver failure tended to use hospital-based palliative care whereas both hospital and community palliative care was used by decedents with motor neurone disease. The largest improvement in access to specialist palliative care over the last ten years was observed for liver failure and COPD followed by renal failure and then heart failure. The numbers of decedents with Huntington’s disease or HIV/AIDS were low and further subgroup analyses were not performed to ensure decedent confidentiality.

### Number of days receiving specialist palliative care in the last year of life

Of the 46 % of the cohort who did access specialist palliative care, the median number of days under specialist palliative care was 25 days (IQR 8–75 days) but this varied greatly by the principal cause of death condition. Decedents with cancer and motor neurone disease received the most specialist palliative care with a median of 30 (IQR 10–81)) and 34 (IQR 12–162) specialist palliative care days respectively. The median number of days receiving specialist palliative care was lower for heart failure (5; IQR 2–15), renal failure (6; IQR 3–17), chronic obstructive pulmonary disease (8; IQR 3–25), liver failure (7; IQR 4–18) and Alzheimer’s disease (5; IQR 3–11).

The temporal distribution of specialist palliative care access days in the last year life was skewed towards the weeks leading up to death. Over 60 % of total days of hospital-based palliative care occurred in the four weeks before death although this did vary by principal cause of death condition. Hospital-based palliative care started a median of 15 (IQR 6–44) days before death for decedents with cancer, 44 (IQR 8–149) days for motor neurone disease and 8 (IQR 4–22) days for decedents with liver failure. Decedents with other cause of death conditions started hospital-based palliative care a median of around 5–6 days before death.

Community-based palliative care tended to be accessed earlier in the last year of life with a median starting date of 62 (IQR 26–137) days before death for cancer, 192 (IQR 19–365) days for Huntington’s disease and 86 (IQR 20–253) days for motor neurone diseases. For the other conditions, the median starting dates ranged from 6 (IQR 3–54) days for Alzheimer’s disease up to 43 (IQR 15–138) days for chronic obstructive pulmonary disease.

### Factors associated with access to specialist palliative care services

Ten years earlier it was reported that decedents with cancer who were not partnered, were living outside a major city and were older than 85 years of age had reduced odds of receiving specialist palliative care. Similar subgroup analyses were performed here ten years later with increased odds of specialist palliative care observed for cancer decedents with a partner (OR 1.1; 95 % CI 1.0–1.3; *p*-value 0.011), female cancer decedents (OR 1.2; 95 % CI 1.1–1.3; *p*-value 0.001), cancer decedents living in a major city compared to very remote (OR 2.8; 95 % CI 1.8–43; *p*-value <0.001) or cancer decedents aged <60 years compared to 90 years (OR 1.8; 95 % CI 1.4–2.3; *p*-value <0.001). It was also observed that cancer decedents living in an aged care facility at time of death had much reduced odds of accessing specialist palliative care compared to decedents living in a private residence (OR 0.2; 95 % CI 0.2–0.3; *p*-value <0.001). A similar subgroup analysis restricted to non-cancer decedents observed that the only social and demographic variable associated with reduced access to specialist palliative care was for those decedents living in a care facility compared to a private residence (OR 0.4; 95 % CI 0.3–0.4; *p*-value <0.001).

However, a disadvantage of these subgroup analyses is that comparisons of specialist palliative care use cannot be made between subgroups and overall statistical power is reduced. To overcome these limitations, two logistic regression models that included all decedents were constructed; the first with all sociodemographic variables and the second with all sociodemographic variables and the underlying cause of death condition. In Adjusted Model 1, all sociodemographic variables remained independently significantly associated with access to specialist palliative care when tested simultaneously using multivariate logistic regression modelling (Table 3). Without taking any of the causes of death into account, higher proportions of decedents accessed specialist palliative care if they were younger, male, had a partner, lived in more urban areas, lived in higher socioeconomic areas and lived in a private residence at time of death.

**Table 3:** The number, proportion and adjusted odds ratios of social and demographic characteristics associated with accessing specialist palliative care (*n* = 12817)

	Accessed any SPC			Adjusted model 1^a^			Adjusted model 2^b^		
	N	%	*p*-value	OR	95 % CI	*p*-value	OR	95 % CI	*p*-value
Age at death (years)									
< 60	1,042	62.9	<0.001	3.3	2.8 − 3.9	<0.001	1.4	1.2 − 1.7	<0.001
60–69	1,207	60.0		2.8	2.4 − 3.3	<0.001	1.3	1.1 − 1.5	0.009
70–79	1,719	53.6		2.4	2.0 − 2.7	<0.001	1.4	1.2 − 1.6	<0.001
80–89	1,595	37.8		1.5	1.3 − 1.8	<0.001	1.1	1.0 − 1.3	0.095
90+	369	21.4		1	ref	−	1	ref	−
Sex									
Male	3,363	48.4	<0.001	1	ref	−	1	ref	−
Female	2,569	43.7		1.1	1.1 − 1.2	0.002	1.2	1.1 − 1.3	<0.001
Partner									
No or unknown	2,646	38.6	<0.001	1	ref	−	1	ref	−
Yes	3,286	55.2		1.2	1.1 − 1.3	<0.001	1.2	1.1 − 1.3	0.001
Accessibility index									
Major cities	4,234	48.2	<0.001	1	ref	−	1	ref	−
Inner regional	896	42.3		0.8	0.7 − 0.9	<0.001	0.8	0.7 − 0.9	<0.001
Outer regional	562	46.2		0.9	0.8 − 1.0	0.061	0.9	0.8 − 1.1	0.266
Remote	167	39.0		0.6	0.5 − 0.8	<0.001	0.6	0.5 − 0.7	<0.001
Very remote	65	27.9		0.4	0.3 − 0.6	<0.001	0.4	0.3 − 0.6	<0.001
IRSD of area of residence									
1 (most disadvantaged)	1,217	43.7	<0.001	1	ref	−	1	ref	−
2	1,298	46.7		1.1	1.0 − 1.2	0.239	1.1	0.9 − 1.2	0.404
3	1,160	44.5		1.1	1.0 − 1.2	0.213	1.0	0.9 − 1.1	0.950
4	1,047	48.1		1.2	1.0 − 1.3	0.015	1.1	0.9 − 1.2	0.345
5 (least disadvantaged)	1,203	49.5		1.2	1.1 − 1.4	0.001	1.1	1.0 − 1.3	0.064
Residence at death									
Private residence	5,472	55.9	<0.001	1	ref	−	1	ref	−
RACF	406	14.9		0.2	0.2 − 0.2	<0.001	0.3	0.2 − 0.3	<0.001
Non-aged care facility	25	15.9		0.2	0.1 − 0.3	<0.001	0.3	0.2 − 0.5	<0.001
Other/unknown	29	20.9		0.3	0.2 − 0.4	<0.001	0.4	0.2 − 0.6	<0.001

*SPC* specialist palliative care (hospital and/or community-based), *IRSD* Index of relative social disadvantage, *RACF* Residential aged care facility, *OR* Odds Ratio

^a^Adjusted model 1 included all social and demographic variables simultaneously; ^b^Adjusted model 2 was identical to Adjusted Model 1 except it also included the principal cause of death conditions (OR shown in results section text)

When the underlying cause of death were also accounted for, the relative impact of sociodemographic variables associated with specialist palliative care was modified (Table 3; Adjusted Model 2). Living in areas of lower socioeconomic status was no longer associated with reduced access to specialist palliative care and the association of age at death was not as marked. Decedents who were living in major cities, were female, had a partner and were living in a private residence at time of death still had increased odds of access to specialist palliative care after adjusting for the cause of death condition.

Using decedents with heart failure as the referent group and adjusting for sociodemographic variables, the relative odds of accessing specialist palliative care in the last year of life was increased for decedents with cancer (OR 10.5; 95 % CI 9.1–12.2), renal failure (OR 1.5; 95 % CI 1.3–1.9), liver failure (OR 2.3; 95 % CI 1.7–3.3) or motor neurone disease (OR 4.5; 95 % CI 3.1–6.6). There was no significant difference in the odds of access to any specialist palliative care between decedents with heart failure and those with chronic obstructive pulmonary disease, Alzheimer disease, Parkinson’s disease, Huntington’s disease or HIV/AIDS, however the small number of decedents in these disease groups reduced the statistical power to detect anything but very large differences.

## Discussion

There has been a significant 3.5 % increase in access to specialist palliative care services in the last year of life at a population level over a ten year period. Most of this increase was driven by increased uptake of palliative care service by people dying of non-cancer related conditions, particularly those with liver failure, COPD and renal failure. This is a positive sign that recommendations from the 2005 WA Government Report and the subsequent Palliative Care model of care endorsed in 2008 are having an impact in the community [24, 25]. The 2005 Palliative Care report highlighted the need to provide increased services for life limiting illness, regardless of diagnosis, and proposed working with existing support organizations of conditions other than cancer. While the 6.5 % increase in people with non-cancer conditions receiving specialist palliative care is encouraging, we must still question why so few people with non-cancer conditions still do not access specialist palliative care.

In our study, the largest proportion of specialist palliative care users in the non-cancer group died with motor neurone disease or liver failure. It was not surprising that decedents with motor neurone disease had increased exposure to specialist palliative care with over 30 % receiving care in the community. The progressive and ultimately fatal trajectory in most people with motor neurone disease makes referral to specialist palliative care a more obvious decision for clinicians. In addition, the not-for profit Motor Neurone Disease Association has been providing palliative care information and care support services to patients and their families since 1983 [26].

In contrast to patients with motor neurone disease, more decedents with liver failure received specialist palliative care in hospital rather than the community. This is likely a reflection of the end-stage liver disease complexity, comorbidity and difficulties with managing symptoms, such as severe ascites, encephalopathy and variceal bleeding, in the community. In addition, there is the uncertain experience of waiting for a liver transplant, the only existing cure which is ultimately only available to a minority of patients [27].

The proportion of decedents with other non-cancer conditions considered amenable to palliative care who received specialist palliative care in the last year of life was relatively lower. Less than 20 % of decedents with the more common life limiting conditions of heart failure, renal failure and chronic pulmonary respiratory disease accessed specialist palliative care in the last year of life - and this care was accessed in the final few weeks. The reasons for the late access to specialist palliative care is likely to be multifactorial and condition specific although it has been observed that, in general, patients with organ failure tend to have less understanding of the disease trajectory compared to patients with cancer [14]. Similar patterns of palliative care in non-cancer conditions are also reported elsewhere with 19 % of heart failure patients accessing palliative care in a US setting and the median time for first palliative care consultation occurring only 21 days before death [28]. Reported barriers to accessing palliative care in heart failure include lack of patient awareness of the life-limiting or progressive nature of their condition, a fear of hospice care not providing sufficient symptom relief [29], uncertain prognosis and disease trajectory [30]. Given the high one year mortality rate for heart failure it has been suggested that end of life preparedness planning should be offered as early as time of diagnosis [31].

Western Australia is the largest state in Australia with a land area of 2.5 million km^2^ (larger than Western Europe), mostly sparsely populated except for the south west corner which includes the capital city, Perth. We observed reduced access to specialist palliative care for decedents living in inner regional and remote areas relative to those living in the major cities, as was observed ten years earlier. However, it does appear that decedents living in outer regional areas now have similar access to specialist palliative care as major urban areas. These finding are encouraging and support a recent report on palliative care services in rural areas of Western Australia that documented the establishment of regional palliative care teams, increased education to palliative care providers, inclusion of social workers into palliative care teams and visiting palliative care specialist services [32].

We noted that specialist palliative care was accessed more often by younger decedents and decedents with partners, regardless of the disease condition. We question whether this is a reflection of societal perceptions that the elderly are less needy in requiring end of life care or whether coming to terms with and dealing with imminent death is less burdensome as one ages? The increased uptake of specialist palliative care in decedents with partners at the time of death highlights the role that the palliative care team plays in supporting carers as well as the dying patient and is likely reflecting an increase in advocacy by carers on behalf of their patients in accessing palliative care.

While this study had the advantage of being large and population based it did have several important limitations. Firstly, we had little information on the severity of the disease condition assigned as being amenable to palliative care or whether the death was truly expected or not. Thus it may not have always been clinically appropriate for the decedent to be referred to palliative care. Secondly, we did not have data to estimate how much of normal care provided by residential aged care facilities could be classified as being palliative in nature, and thus, we may be underestimating the level of palliative care being delivered in these facilities. And lastly, we are also likely to be underestimating the access to specialist palliative care in rural areas because we relied on community based palliative care data from a single provider that is focused in urban areas. Measurement of improvements in access for patients living in rural and remote areas will require additional data to be conclusive. We also acknowledge that life limiting conditions that may benefit palliative care are likely to change over time, for example, HIV/AIDS. These temporal changes need to be considered when interpreting findings.

## Conclusion

Encouraging findings are reported for improving palliative care access in rural and remote areas. Importantly, we have also shown a small but significant increase in access to specialist palliative care services in Western Australia, specifically in patients dying with non-cancer conditions. However, while palliative care peak bodies continue to espouse that palliative care should be available to all who need it regardless of diagnosis, further work is needed. In particular, methods to evaluate improvements such as we have presented here are essential to document progress and are a first step in providing much needed evidence to support changes in funding and clinical practice to support palliative care for all.

## Availability of data and materials

The datasets supporting the conclusions of this article are available from the corresponding author provided that written approval from the relevant data custodians and Western Australian Department of Health Human Research Ethics Committee has been obtained. Approvals must be obtained within seven years of last use of the datasets after which the data will be destroyed according to the original conditional HREC approval.

## Additional file

Additional file 1: Table S1. ICD-10-AM codes used to identify the 10 cause of death conditions in the cohort. (DOCX 13 kb)
