# Supplementary material for: A retrospective population based cohort study of access to specialist palliative care in the last year of life: who is still missing out a decade on?
Source: BMC Palliat Care. 2016 May 10;15:46. doi: 10.1186/s12904-016-0119-2 (PMC4862038; doi:10.1186/s12904-016-0119-2)
Supplement: Additional file 1: Table S1. — ICD-10-AM codes used to identify the 10 cause of death conditions in the cohort. (DOCX 13 kb) [file 12904_2016_119_MOESM1_ESM.docx]

Table S1. ICD-10-AM codes used to identify the 10 cause of death conditions in the cohort.

| **Condition** | **ICD-10-AM codes** |
| --- | --- |
| Cancers | All codes beginning with C, D1 D2 D3 D4 |
| Heart failure | I500 I501 I509 I110 I130 I132 |
| Renal failure | N180 N188 N189 N19 E1023 E1123 E1323 I120 I131 I132 |
| Liver failure | K704 K711 K721 K729 |
| COPD | J40 J410 J414 J418 J42 J430 J431 J432 J438 J439 J440 J441 J448 J449 J47 |
| Alzheimer's disease | G300 G301 G308 G309 |
| Motor neurone diseases | G122 |
| Parkinson's disease | G20 |
| Huntington's disease | G10 |
| HIV/AIDS | B20 B21 B22 B23 B24 |

COPD = Chronic Obstructive Pulmonary Disease
